# Can local application of Tranexamic acid reduce post-coronary bypass surgery blood loss? A randomized controlled trial

**DOI:** 10.1186/1749-8090-4-25

**Published:** 2009-06-18

**Authors:** Hosam Fawzy, Elsayed Elmistekawy, Daniel Bonneau, David Latter, Lee Errett

**Affiliations:** 1 Division of Cardiovascular and Thoracic Surgery, St. Michael's Hospital, University of Toronto, 30 Bond Street, Toronto, Ontario M5B 1W8, Canada

## Abstract

**Background:**

Diffuse microvascular bleeding remains a common problem after cardiac procedures.

Systemic use of antifibrinolytic reduces the postoperative blood loss.

The purpose of this study was to examine the effectiveness of local application of tranexamic acid to reduce blood loss after coronary artery bypass grafting (CABG).

**Methods:**

Thirty eight patients scheduled for primary isolated coronary artery bypass grafting were included in this double blind, prospective, randomized, placebo controlled study.

Tranexamic acid (TA) group (19 patients) received 1 gram of TA diluted in 100 ml normal saline. Placebo group (19 patients) received 100 ml of normal saline only. The solution was purred in the pericardial and mediastinal cavities.

**Results:**

Both groups were comparable in their baseline demographic and surgical characteristics. During the first 24 hours post-operatively, cumulative blood loss was significantly less in TA group (median of 626 ml) compared to Placebo group (median of 1040 ml) (P = 0.04). There was no significant difference in the post-op Packed RBCs transfusion between both groups (median of one unit in each) (P = 0.82). Significant less platelets transfusion required in TA group (median zero unit) than in placebo group (median 2 units) (P = 0.03). Apart from re-exploration for excessive surgical bleeding in one patient in TA group, no difference was found in morbidity or mortality between both groups.

**Conclusion:**

Topical application of tranexamic acid in patients undergoing primary coronary artery bypass grafting led to a significant reduction in postoperative blood loss without adding extra risk to the patient.

## Introduction

Coagulopathy remains a common problem after coronary artery bypass Grafting (CABG) using cardiopulmonary bypass (CPB). It results from many factors like thrombocytopenia, acquired platelet dysfunction, clotting factors loss, free heparin, and increased fibrinolysis [1-3]. Lemmer and Colleagues [4] found that extracorporeal circulation results in significant fibrinolysis, as reflected by increased concentrations of plasmin and fibrin degradation products (FDP), both of which have deleterious effects on platelet function. Re-exploration for bleeding following cardiac surgery with CPB was reported to be in the range of 2–7%. Of these, 50–80% was found to be medical rather than surgical bleeding [5]. Fibrinolysis was found to be responsible for 25–45% of significant post bypass bleeding [6]. Many antifibrinolytic agents have been used to diminish post-bypass bleeding. These include ε Aminocaproic acid [5], aprotinin [7], and Tranexamic acid (TA) [8]. Tranexamic acid has been found to bind to lysine binding sites of plasmin and plasminogen. Saturation of these sites displaces plasminogen from the fibrin surface thus inhibiting fibrinolysis [9]. TA has been used both systemically and topically. Intravenous TA administration increased the risk of thromoembolic complications and consequently early graft closure in coronary artery bypass grafting [10]. When used topically, TA was found effective in controlling bleeding in patients with hemorrhagic diathesis and in patients who were being treated with anticoagulants pre-operatively. Topical TA has also been successfully used in controlling bleeding in bladder, gynaecologic, oral, and otolaryngeal surgeries [11-13].

This prospective, double-blind, randomized, placebo-controlled study was designed to investigate the effect of topical TA in reducing postoperative blood loss after coronary artery bypass Grafting.

## Methods

With institutional ethics committee approval, all patients scheduled for primary isolated elective CABG at North West Armed Forces Hospital, Tabuk, Saudi Arabia, during the period from March 2004 to November 2005, were scrutinized for eligibility enrolment. Our exclusions criteria included patients who had combined procedure; redo surgery, bleeding diathesis (Haemophilia or platelet count < 100 × 10^9 ^L^-1^), renal failure (Creatinine > 160 mg/dl), known allergy to TA, recent (<7 days before surgery) intake of anti-platelets (e.g. Aspirin, non-steroidal anti-inflammatory drugs) or Heparin administration within 48 hours of operation.

Thirty eight patients met the requirements for inclusion, and informed consent was obtained from all of them. The patients were randomly allocated into one of the two groups. Group I (TA group) included 19 patients who received 1 gm of TA diluted in 100 ml normal saline. Group II (Placebo group) included 19 patients who received 100 ml normal saline as placebo. The solution was purred in the pericardial and mediastinal cavities before closure of the strenotomy while clamping the chest tubes. These clamps were released once the closure of the strenotomy was completed.

The study was carried out as a prospective randomized, double blind investigation. Randomization was carried out with random-number tables by a research pharmacist who prepared the two solutions in two identical bottles and delivered to the operating theatre. Neither the surgeon, assistant, anaesthetist, scrub nurse nor the perfusionist knew the composition of the solution administered. Only two cardiac surgeons were responsible for the surgical haemostasis.

The anaesthetic management and conduct of CPB were standardized. The patients were premedicated using Nitrozepam 0.1 mg/kg tablet the night of the operation, and morphine 0.15 mg/kg intramuscular half an hour before operation. Induction was done using Fentanyl 2–5 ug/kg, Propofol 1–2 mg/kg, and Pancronium 0.1 mg/kg. Anaesthesia was maintained by using Sevoflurane 0.5–1%, Pancronium 0.06 mg/kg, Fentanyl 1–2 ug/kg during cardiopulmonary bypass time. All patients received Heparin 300 units/Kg before CPB to achieve target activated clotting time (ACT) of ≥ 480 seconds. During CPB, extra heparin was given as needed to maintain the target ACT. After separation from CPB, heparin was reversed using protamine sulphate in the dose of 1 mg/100 units of heparin to achieve target ACT 80–120 seconds.

After the patient was transferred to the intensive care unit (ICU), continuous low grade suction (10–15 cm H_2_O) was applied together with periodic milking of the drains. Haemoglobin level (Hb), Hematocrite value (Hct %), Platelet count, International Normalized Ratio (INR), Partial Thromboplastin time (PTT), and Fibrinogen level were measured before the operation and when the patients arrived at the intensive care unit. The drainage of the chest tubes was measured hourly and were removed when the total drainage volume of 80–100 ml over the previous12 hours and of serous color. Uniform transfusion protocol was applied to all patients. Blood and blood components were administered only when the hematocrite level < 24% or the haemoglobin level ≤ 8.0 gm/dL in the postoperative period. Shed mediastinal blood was not transfused into any patient during this study.

Beside patients' demographics, the numbers of grafts, left internal mammary artery (LIMA) use, cross clamp time, duration of CPB, incidence of reoperation for bleeding, ICU stay and the length of hospital stay (LOS) were recorded for all patients.

### Statistical Analysis

Parametric data were analysed using 2-tailed unpaired student t test, and presented as mean ± standard deviation. Data analysis for blood loss and requirements for blood products was done using non-parametric tests (Mann-Whitney Rank Sum test) using Sigma Stat (v 3.5, Systat Software San Jose, California, USA), and expressed as median and range. Values of p less than 0.05 were considered significant.

## Results

Both groups were comparable with respect to baseline demographic data and there was no statistically significant difference in the prevalence of risk factors between both groups as shown in Table 1.

**Table 1:** Patients' Demographics.

	**TA (n = 19)**	**Placebo (n = 19)**	***P*-Value**
Age (y)	55 ± 11	60 ± 7	0.17
M/F	18/1	18/1	1.0
Wt. (Kg)	73 ± 15	71 ± 14	0.74
BMI	27 ± 4	27 ± 4	1.0
Smoking	6 (31.5%)	6 (31.5%)	1.0
Diabetes	7 (37%)	10 (53%)	0.42
Hypertension	7 (37%)	12 (63%)	0.17
Hyperchol	3 (16%)	0 (0%)	0.08
COPD	1 (5%)	1 (5%)	1.0
RF	0 (0%)	1 (5%)	0.33
PVD	3 (16%)	0 (0%)	0.08
Previous MI	4 (21%)	6 (31.5%)	0.42
LVEF	48 ± 10	46 ± 10	0.49
Euroscore	2.3 ± 1.7	2.5 ± 1.6	0.58

Values are means ± SD or No.(%) where shown.

M/F: Male/Female

Wt.: Weight

BMI: Body Mass Index

COPD: Chronic Obstructive Pulmonary Disease

RF: Renal Failure

Hyperchol: Hypercholesterolmia

PVD: Peripheral Vascular Disease

MI: Myocardial Infarction

LVEF: Left Ventricular Ejection Fraction

**Figure 1:**
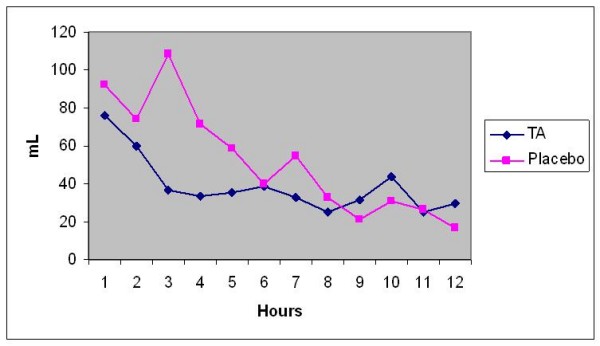
**Post-op Blood Loss/Hour**. Our study demonstrates that pouring of one gram of TA into the pericardial cavity after CABG, significantly reduced post-operative blood loss in the first 24 hours after surgery (37%) with the maximum reduction at the 3rd hour (66%).

**Figure 2:**
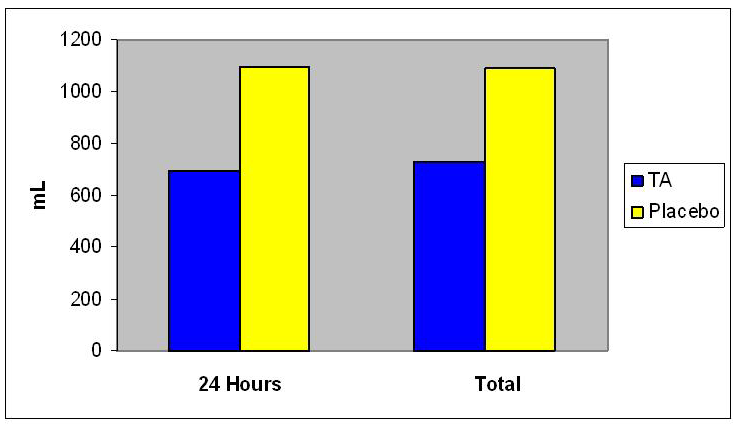
**Post-op Blood Loss/24 hours and total**. Chest tube drainage in the first 24 hours was significantly less in TA group (median of 626 ml) than in the placebo group (median of 1040 ml) (P = 0.04). This represented about 37% decrease in the blood loss. The median total post-operative chest tube drainage was 656 ml (range 248–2105) in TA group and 1056 ml (range 210–3010) in the placebo group, which represents 32% reduction in total bleeding.

The two groups were matched with regard to intraoperative data; use of left internal mammary artery, average number of distal anastmoses, cross clamp time, cardiopulmonary bypass (CPB) time and duration of the operation (Table 2). There was no bias in the distribution of the anaesthesiologist and surgeons between the two groups. Pre and postoperative haemoglobin concentrations, hematocrit concentrations, platelet counts, international normalized ratio, partial thromboplastin times and fibrinogen level were not significantly different between the two groups (Tables 3 &4). Chest tube drainage in the first 24 hours was significantly less in TA group (median of 626 ml) than in the placebo group (median of 1040 ml) (P = 0.04). This represented about 37% decrease in the blood loss. The median total post-operative chest tube drainage was 656 ml (range 248–2105) in TA group and 1056 ml (range 210–3010) in the placebo group, which represents 32% reduction in total bleeding (Figures 1 &2).

**Table 2:** Intra-operative Data.

	**TA**	**Placebo**	***P*-Value**
Graft/pt	2.8 ± 1.1	2.9 ± 1.0	0.64
LIMA	100%	100%	1.0
CX time (min)	72 ± 17	75 ± 20	0.09
TBT (min)	100 ± 28	97 ± 28	0.07

Values are means ± SD or (%) where shown.

LIMA: Left Internal Mammary Artery.

CX Time: Cross Clamp time.

TBT: Total Bypass time.

**Table 3:** Pre-operative Hematological Profile.

	**TA**	**Placebo**	***P*-Value**
Platelets	265 ± 119	156 ± 71	0.77
Hb (gm/dl)	14 ± 1.0	14 ± 1.3	0.08
Hct (%)	39 ± 2.8	41 ± 3.7	0.15
INR	1.1 ± 0.3	1.0 ± 0.2	0.23
PTT	31.2 ± 4.8	33.6 ± 3.5	0.34
Fibrinogen	362 ± 163	438 ± 158	0.46

Values are means ± SD where shown.

Hb: Hemoglobin.

Hct: Hematocrite Value.

INR: International Normalized Ratio.

PTT: Partial Thromoplastin Time.

**Table 4:** Post-operative Hematological Profile.

	**TA**	**Placebo**	***P*-Value**
Platelets	192 ± 64	196 ± 68	0.86
Hb (gm/dl)	10 ± 1.3	10 ± 1.3	0.39
Hct (%)	29 ± 3.8	30 ± 3.9	0.39
INR	1.3 ± 0.3	1.2 ± 0.4	0.18
PTT	41.2 ± 5.4	45.6 ± 7.4	0.22
Fibrinogen	239 ± 49	359 ± 202	0.15

Values are means ± SD where shown.

Hb: Hemoglobin.

Hct: Hematocrite Value.

INR: International Normalized Ratio.

PTT: Partial Thromoplastin Time.

**Figure 3:**
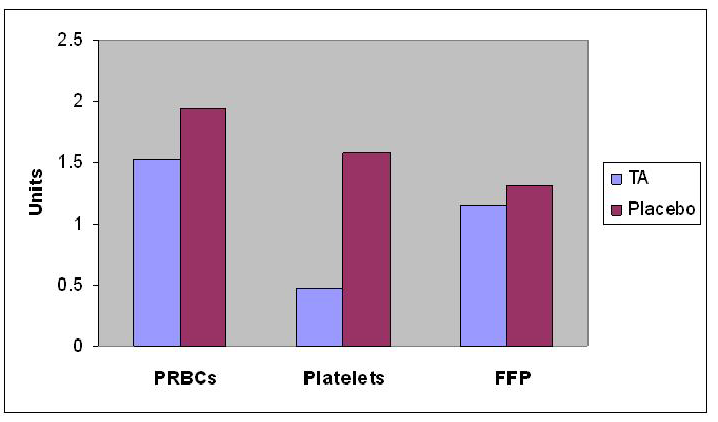
**Post-op Blood Products Transfusion**. There was no significant difference in the post-op packed red blood cells (PRBCs) transfusion between both groups (median of one unit in each) (P = 0.82). Also there was no significant difference in regard Fresh Frozen Plasma (FFP) transfusion between both groups (Median of zero unit in TA vs. two units in placebo) (P = 0.42). Significant more platelets transfusion required in the Placebo group (median 2 units) than in TA group (median zero unit) (P = 0.03)

There was no significant difference in the post-op packed red blood cells (PRBCs) transfusion between both groups (median of one unit in each) (P = 0.82). Also there was no significant difference in regard Fresh Frozen Plasma (FFP) transfusion between both groups (median of zero unit in TA vs. two units in placebo) (P = 0.42). Significant more platelets transfusion required in the Placebo group (median 2 units) than in TA group (median zero unit) (P = 0.03) (Figure 3). Troponin I level recorded a lower level in TA group (2.4 ± 3.8) than Placebo group (4.7 ± 2.0) (P = 0.68). There was no post-op myocardial infarction encountered in either group. Although there was no difference in intubation time between both groups, Placebo group patients stayed significantly longer in ICU (49 ± 20 Hrs) than the TA patients (29 ± 26) (P = 0.02). Total length of Hospital stay was comparable in both groups (Table 5). Apart from re-exploration for excessive surgical bleeding in one patient in the TA group (due to branch bleeding of one of the grafts), no difference was found in morbidity or mortality between the two groups. There were no deaths in either group.

**Table 5:** Post-operative data.

	**TA**	**Placebo**	*P*-Value
Troponin I	2.4 ± 3.8	4.7 ± 2.0	0.68
(mmol/L)			
Intub (Hrs)	14 ± 7	17 ± 12	0.52
ICU (Hrs)	29 ± 26	49 ± 20	0.02*
LOS (Days)	7.5 ± 3	7.8 ± 2.0	0.68

Values are means ± SD where shown.

Intub: Intubation time.

ICU: Intensive Care Unit.

LOS: Length of Hospital Stay.

## Discussion

Intravenous use of aprotinin, tranexamic acid and aminocaproic acid has proved to be effective in decreasing post-operative bleeding in both primary and redo coronary artery bypass grafting. However, the risk of death is consistently higher with use of aprotinin than with tranexamic acid or aminocaproic acid [14].

In the large International Multi-center Aprotinin Graft Patency Experience (IMAGE) study, no statistically significant difference in graft occlusion could be found between patients given Aprotinin and controls when the results were adjusted for risk factors known to be associated with graft failure [15]. Therefore, the benefit of antifibrinolytic agents must be always weighted against a possible increase risk of thrombo-embolic complications that might lead to early graft closure. Moreover, these patients are at increase risk of cerebral, pulmonary, mesenteric and retinal thrombosis.

Following the success of systemic use of Aprotinin and Tranexamic acid in controlling post-CABG bleeding, trials for topical use were initiated by Tatar et al [16] in 1993, who reported reduced post-operative blood loss and the need for transfusion after topical Aprotinin use in CABG patients. Similar results were described by O'Regan and his colleagues [17] and Khalil et al [18]. This encouraged De Bonis group [19] in 2000 to start topical use of TA. They conducted a prospective randomized, double blinded study on 40 patients who had primary CABG. They reported blood loss significantly diminished by 36% during the first 3 hours but not during the following 21 hours after operation compared to the placebo group. Our study demonstrates that pouring of one gram of TA into the pericardial cavity after CABG, significantly reduced post-operative blood loss in the first 24 hours after surgery (37%) with the maximum reduction at the 3rd hour (66%). Abdul Azem and his colleagues [20] in 2006 described similar results in 100 patients underwent various open heart procedures. In contradictory to our findings, Yasim and his group [21] did not find a statistically significant reduction in post-operative bleeding after topical application of Aprotinin and Tranexamic acid.

In our study the overall post-operative bleeding was modest in both groups and this could be explained by the tight inclusion criteria, meticulous surgical hemostasis, normothermic CPB and the use of LIMA alone as a pedicle graft in all patients. Therefore, despite the significant reduction of post-operative bleeding in TA group, the effect of its topical use might be masked by those factors. A greater effect of topical TA in reducing blood loss could be possibly evident in more prolonged and complex procedures with a higher risk of bleeding.

The use of blood products was not significantly different between both De Bonis' groups [19], while we had a significant reduction only in platelet transfusion and Abdul Azm [20] had a significant reduction only in packed cells use in TA group. This difference in reports might be explained by the difference in number of patients, type of operations and transfusion protocols used in each study. The advantage of topical use of antifibrinolytic drugs after open heart surgery, in reducing post-operative bleeding and transfusion requirement, needs further clinical trials using larger number of patients [22].

## Limitation of the study

We did not measure the level of TA in the serum or in the pericardial cavity. The number of the patients was relatively small and the inclusion criteria with strictly tight. Larger multi-center prospective control trials using larger dose of TA are needed to address its topical effect in procedures with higher risk of bleeding.

## Conclusion

Topical use of Tranexamic acid into the pericardial cavity in patients undergoing primary coronary artery bypass grafting significantly reduces postoperative bleeding and platelet transfusion without adding extra risk to the patient. Consequently Tranexamic acid could be advocated for routine use topically in patients undergoing coronary artery bypass grafting.

## Competing interests

The authors declare that they have no competing interests.

## Authors' contributions

HF participated in the study design, in analyzing the data, writing, reviewing and submitting the manuscript. EM conceived of the study, participated in its design and carried out the coordination, collecting the data. DB participated in reviewing the manuscript. DL participated in reviewing the manuscript. LE participated in reviewing the manuscript. All authors read and approved the final manuscript.
